# History of Inuit Community Exposure to Lead, Cadmium, and Mercury in Sewage Lake Sediments

**DOI:** 10.1289/ehp.7985

**Published:** 2005-05-31

**Authors:** Mark H. Hermanson, James R. Brozowski

**Affiliations:** 1 Department of Chemistry, University of Pennsylvania, Philadelphia, Pennsylvania, USA; 2 Department of Soil, Water, and Climate, University of Minnesota, St. Paul, Minnesota, USA

**Keywords:** cadmium, Canada, exposure, history, Inuit, lakes, lead, mercury, sediments, sewage

## Abstract

Exposure to lead, cadmium, and mercury is known to be high in many arctic Inuit communities. These metals are emitted from industrial and urban sources, are distributed by long-range atmospheric transport to remote regions, and are found in Inuit country foods. Current community exposure to these metals can be measured in food, but feces and urine are also excellent indicators of total exposure from ingestion and inhalation because a high percentage of each metal is excreted. Bulk domestic sewage or its residue in a waste treatment system is a good substitute measure. Domestic waste treatment systems that accumulate metals in sediment provide an accurate historical record of changes in ingestion or inhalation. We collected sediment cores from an arctic lake used for facultative domestic sewage treatment to identify the history of community exposure to Pb, Cd, and Hg. Cores were dated and fluxes were measured for each metal. A nearby lake was sampled to measure combined background and atmospheric inputs, which were subtracted from sewage lake data. Pb, Cd, and Hg inputs from sewage grew rapidly after the onset of waste disposal in the late 1960s and exceeded the rate of population growth in the contributing community from 1970 to 1990. The daily per-person Pb input in 1990 (720,000 ng/person per day) exceeded the tolerable daily intake level. The Cd input (48,000 ng/person per day) and Hg input (19,000 ng/person per day) were below the respective TDI levels at the time.

The presence of toxic heavy metals in the Arctic is evidence of long-range atmospheric transport (LRAT) of emissions from industrial and urban areas in temperate regions. Aquatic, marine, and terrestrial organisms—the “country foods” of the Inuit—accumulate these metals from the environment, subjecting native populations to high levels of exposure.

Lead, cadmium, and mercury are all naturally occurring but have no human nutritive function and are considered to be toxins. LRAT has moved atmospheric industrial emissions of these metals around the world for more than a century. The atmospheric residence times of Pb (~ 9 days), Cd (~ 4 days) (Pilgrim and Hughes 1994), and Hg (~ 4 days for particulate and 223 days for gas) (Šakalys and Kvietkus 2002) are long enough for them to reach the Arctic, where they are enriched [Arctic Monitoring and Assessment Program (AMAP) 1998; Hermanson 1991, 1998; Outridge et al. 2002] and are of particular concern because of possible human health effects (Chan et al. 1995; Pacyna 1995). The broad geographic distribution of these toxins has led to efforts to reduce atmospheric emissions, particularly with Pb because of its wide range of industrial uses and addition to gasoline worldwide (Nriagu 1990). Gasoline Pb content declined significantly in North America in the 1970s and 1980s (e.g., from 0.53 g/L in 1974 to 0.026 g/L in 1988 in the United States) [U.S. Department of Interior, Bureau of Mines (USDIBM) 1972, 1982]. In Canada during the late 1980s, however, Pb emissions were still 22 times those of Cd and 48 times those of Hg (Allen 1996). Pb emissions in the United States and Canada in the late 1980s were mostly from industrial processes and waste treatment (AMAP 1998). Cd emissions from nonferrous metal smelters have been reduced, shifting the primary anthropogenic emissions to fossil fuel combustion in the United States and industrial processes in Canada (AMAP 1998; Nriagu 1979; Pacyna 1995; Pilgrim and Hughes 1994; Skeaff and Dubreuil 1997). Hg emissions from nonferrous metal smelters have been reduced to the point that refuse incineration and fossil fuel combustion—particularly coal—are now considered to be the major atmospheric sources (AMAP 1998; Sunderland and Chmura 2000).

Various investigations have identified Inuit exposure to these metals (Dewailly et al. 2001; Dietz et al. 1996), but community exposure history has never been investigated because early data were never collected. As a result, changes in Pb, Cd, and Hg exposures in an Inuit population from reduced or altered emissions are not known.

Our research objective is to identify the history of Pb, Cd, and Hg exposure to the average individual in an Inuit community by measuring inputs of these metals to sewage lake sediments. Many small arctic communities rely on lakes, ponds, or lagoons for natural, facultative treatment of domestic wastes. Because Pb, Cd, and Hg concentrations in urine and feces are considered to be good indicators of exposure to these metals (Bardoděj et al. 1985; Bederka et al. 1985; Choudhury et al. 2001; Claeys-Thoreau et al. 1987; Engqvist et al. 1998; Iwao et al. 1981; Kjellström et al. 1978; Lauwerys et al. 1994; Müller et al. 1993; Sandborgh-Englund et al. 1998; Schouw et al. 2002; Tsuchiya and Iwao 1978), changing metal inputs to sediment in these lakes over time should be an estimate of the exposure history of the contributing community when background effects are considered.

## Materials and Methods

### Study site.

**Figure 1:**
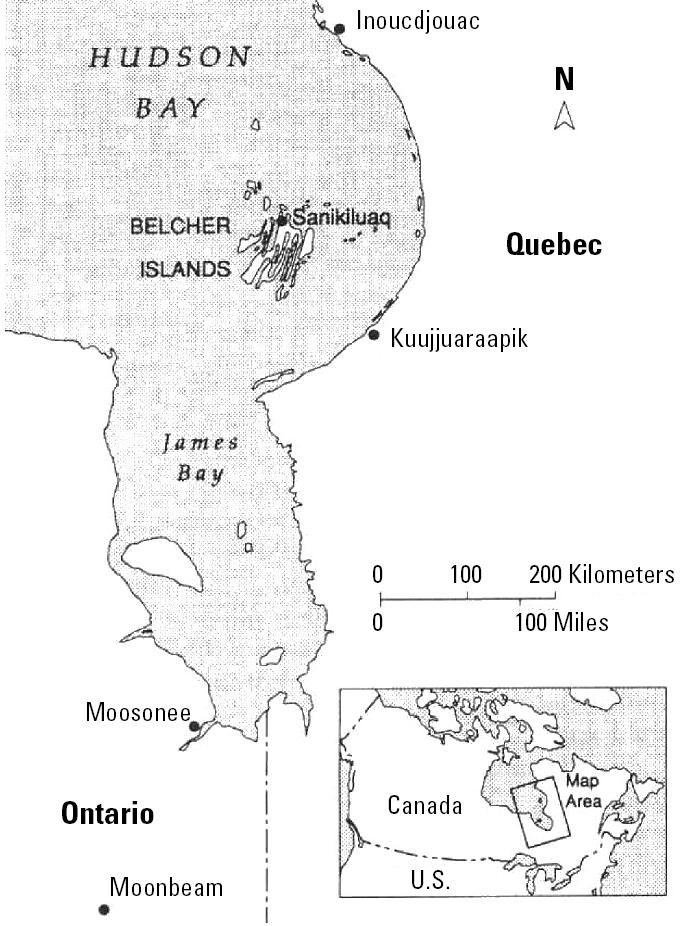
Map of the Hudson Bay area. Reprinted from Hermanson (1991) with permission from the American Chemical Society.

The study site is the Hamlet of Sanikiluaq on the Belcher Islands in southeastern Hudson Bay, Canada (Figure 1). At the time of sampling, about 550 persons, mostly Inuit, lived in the hamlet. The community has a heavy reliance on country foods that are known to be a contaminant source (Wein et al. 1996). No industrial or agricultural sources of Pb, Cd, or Hg are known to exist on the islands. Local activities are not considered to be a source of anthropogenic Pb to the environment other than use of Pb shot for hunting.

**Figure 2:**
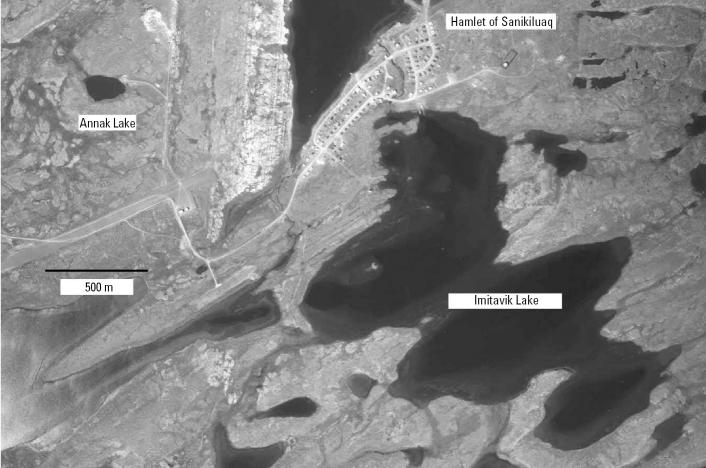
Annotated air photo of the study site. From National Air Photo Library, Canada (1982); reprinted with permission.

We sampled sediment cores collected from two lakes near Sanikiluaq. Annak has been used for disposal of raw domestic waste from the community since about 1968; Imitavik is located 1.5 km to the southeast and is immediately south of the community (Figure 2). The whole-lake focus-corrected flux of Pb, Cd, and Hg in a given period to Annak in excess of that observed at Imitavik reflects the exposure of Sanikiluaq residents to metals in products ingested and inhaled, including food and cigarette smoke—two well-documented metals sources (Benedetti et al. 1992, 1994; Dietz et al. 1996; Gamberg and Scheuhammer 1994; Luoma et al. 1995; Rey et al. 1997; Rickert and Kaiserman 1994; Scheuhammer et al. 1998).

Annak is a small lake (2-ha surface area, 4.5-m maximum depth). Like all small lakes in the area, it is very well mixed by strong surface winds and never stratifies thermally. Nutrients from wastewater have made it eutrophic (open-water pH ~ 10) and anaerobic under ice. Wastewater inputs are quickly dispersed throughout the lake during the open-water season. During winter, wastewater freezes on the eastern shore, entering the lake during thaw in May. Surface sediments, composed mostly of dead phytoplankton, are about 70% organic (Hermanson 1990, 1998). The treatment system is facultative—both aerobic and anaerobic—because of the diel photosynthetic cycle in summer. No engineering structures control its operation. It has no influent streams: its source water is seepage from a small drainage area (42 ha) and wastewater. The metals deposited into the lake are from background, atmosphere, and sewage. Because there are no local industrial or agricultural sources of Pb, Cd, or Hg input to the Sanikiluaq waste stream, excess inputs relative to Imitavik are assumed to be associated with human waste.

Only human waste was discharged to Annak in “honey bags” until 1983. A community-wide remodeling project starting in 1983 transformed the system to tank collection of all household waste. Tanks are now pumped regularly, with the waste transferred to Annak. We assume that household wastes, including cleaning and personal care products, are not significant sources of Pb, Cd, or Hg. Table 1 shows that the estimated fraction of inputs of these products, based on literature values, is not > 1.1% for all categories and each metal except Cd in laundry powder (8.9%).

Imitavik is a multiple basin lake (141-ha surface area, 17.5-km2 drainage basin) fed by many small surface drainage catchments to the south. It is mesotrophic: surface sediments are approximately 25% organic matter and the water pH is approximately 6.5 all year.

### Methods.

In 1993 three sediment cores were collected each from Annak and Imitavik and composited to form one core from each lake. Annak cores were collected from maximum water depth in the lake. Imitavik cores were retrieved near the deepest part of the north basin in water approximately 5.5 m deep. The Imitavik collection area is isolated from most direct drainage basin inputs, so the observed Pb, Cd, and Hg inputs greater than natural fluxes are considered atmospheric in origin. The core collection areas in both lakes are near sites where cores were collected in previous lake sediment studies (Hermanson 1990, 1991, 1993).

Cores were sectioned at 1-cm intervals from the surfaces to a depth of 20 cm and at 2-cm intervals from there to the core bottoms, which exceeded a depth of 40 cm. Sections from a given depth in each of the three cores from a lake were combined to form the composite. Each composite section was analyzed for 210Pb, 137Cs, porosity, and loss on ignition—a surrogate measure for organic carbon. Dating and sedimentation rates were calculated from Pb-210 activity in sediment layers and the constant rate of supply model (Appleby and Oldfield 1978). These values have been reported previously (Hermanson 1998).

A 0.5-g sample of each composite section was digested using 50% nitric acid with microwave heating. Pb and Cd were analyzed from the digestate using graphite furnace atomic absorption spectrophotometry (AAS); Hg was analyzed by manual cold vapor AAS. Accuracy (recovery) from analysis of National Institute for Standards and Technology Standard Reference Material 2704 was 99.9% for Pb, 97.1% for Cd, and 95.0% for Hg. Precision (relative percent difference) was 1.7, 5.4, and 8.6%, respectively. Detection limits were 3.6, 0.16, and 23 ng/g. This digestion includes only those metals associated with organic matter in sediment and contains no mineral fractions.

Fluxes of Pb, Cd, and Hg to individual sections of cores (in nanograms per square centimeter per year) are products of particle deposition rates calculated from Pb-210 (in grams per square centimeter per year) and contaminant concentrations on those particles (in nanograms per gram). Calculating fluxes accounts for changes to sedimentation rates, a particular issue in Annak (Hermanson 1990, 1998). The calculations are converted to whole-lake deposition rates using focus factors (FFs) to correct for postdepositional sediment movement within the lake: the sedimentation rates and metals fluxes thus apply anywhere in the lake. FFs are a comparison of observed Pb-210 inventory in the sediment core to the established focus corrected input of 0.25 becquerel/g (Hermanson 1990). FFs were 2.91 for Imitavik and 1.74 for Annak (Hermanson 1998).

Our goal was to estimate changes in the per-person daily metal excretion in the community over time and compare that with tolerable daily intakes (TDIs) at the time of sampling. We calculated a 1-year input for each metal to Annak for 1970, 1980, and 1990 using the standard units of measure (nanograms per square centimeter per year), shown in Table 2. The background amounts from Imitavik for the same years were subtracted from the Annak values. The net amount for each metal is the total community human contribution to Annak resulting from urinary and fecal excretion. We calculated the average per-person daily contribution (excretion) by dividing these values for each of the 3 years by the community population at the time and number of days in the year.

## Results and Discussion

### Imitavik Lake.

To understand the community exposure recorded in Annak, we need to identify historic atmospheric metal inputs to the area measured in Imitavik.

**Figure 3:**
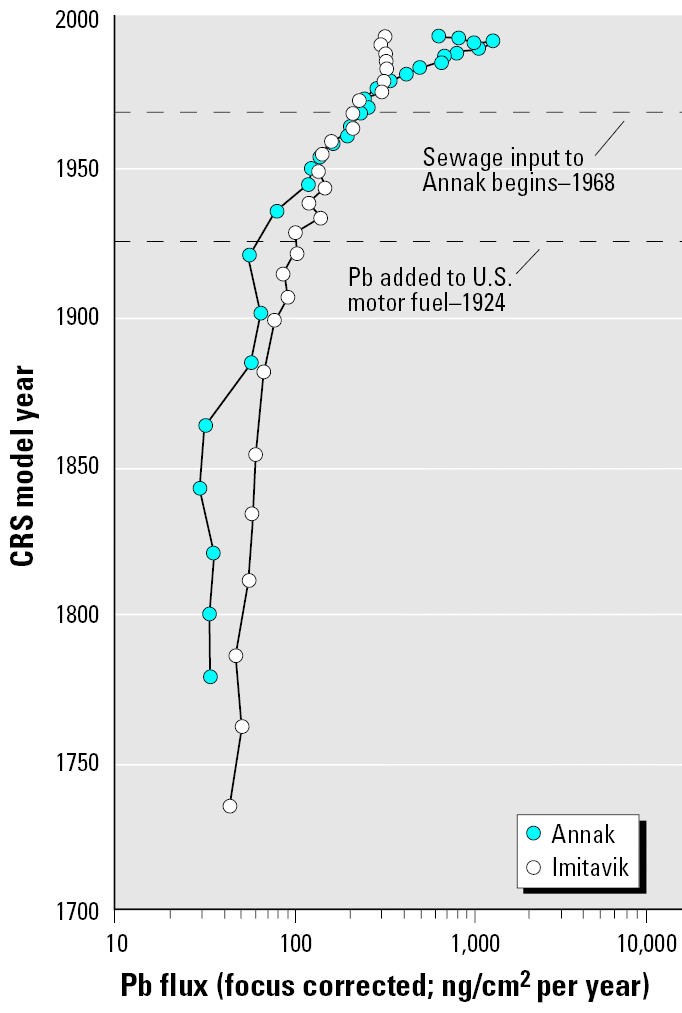
Pb inputs to Imitavik and Annak since the 1700s. CRS, constant rate of supply.

The input of Pb to Imitavik in the 1700s was about 50 ng/cm2 per year (Figure 3) and grew exponentially during the Industrial Revolution from about 64 ng/cm2 per year in 1880 to 325 ng/cm2 per year in 1981. After 1981 the input stopped growing and appears to have declined slightly to about 300 ng/cm2 per year in 1993. Analysis of stable Pb isotopes in the same Imitavik sediment core shows that the area has received a mix of U.S. and Canadian industrial and urban Pb via LRAT since the 1800s (Outridge et al. 2002).

**Figure 4:**
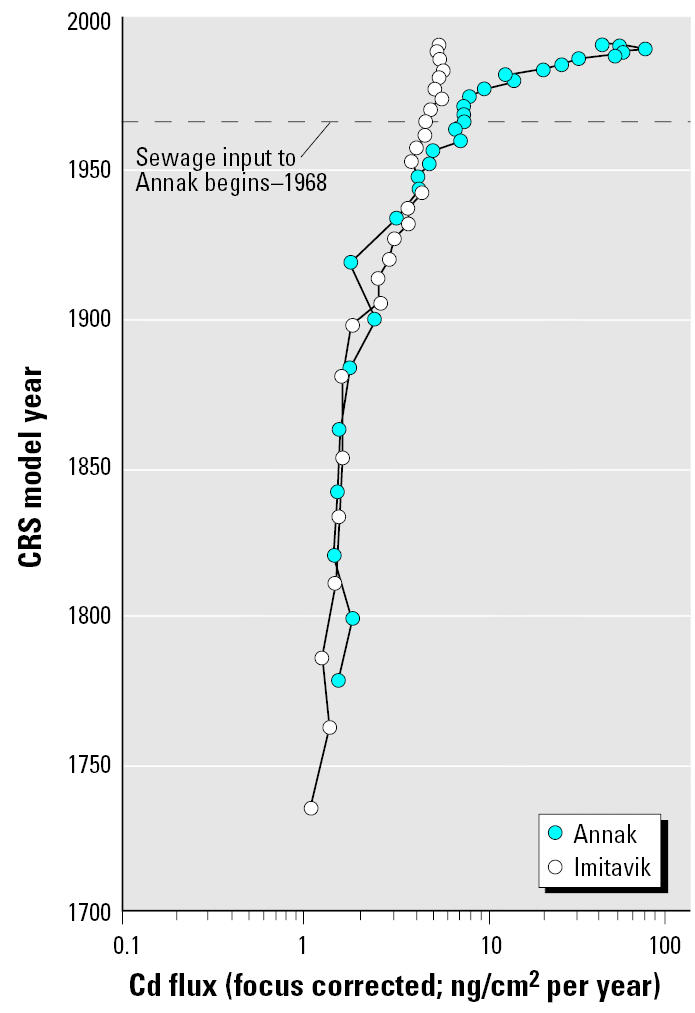
Cd inputs to Imitavik and Annak since the 1700s. CRS, constant rate of supply

Cd deposition to Imitavik was about 1.1 ng/cm2 per year in the early 1700s and increased slowly until 1900, when the total deposition was about 1.8 ng/cm2 per year (Figure 4). Soon after 1900 the annual rate of increase grew rapidly, reaching about 4 ng/cm2 per year near the end of World War II. The Cd deposition growth rate was rapid enough that it doubled between 1900 and 1930, half the time required for doubling of Pb inputs. Unlike Pb, however, the Cd input had not doubled again as of 1993. In the 1940s Cd deposition appears to have stopped increasing until about 1952. From then until 1984, Cd inputs increased at an annual average rate similar to that of 1900–1940 until it reached 5.4 ng/cm2 per year in 1984. Between 1984 and 1993, the Cd deposition appeared to decline approximately 5%.

**Figure 5:**
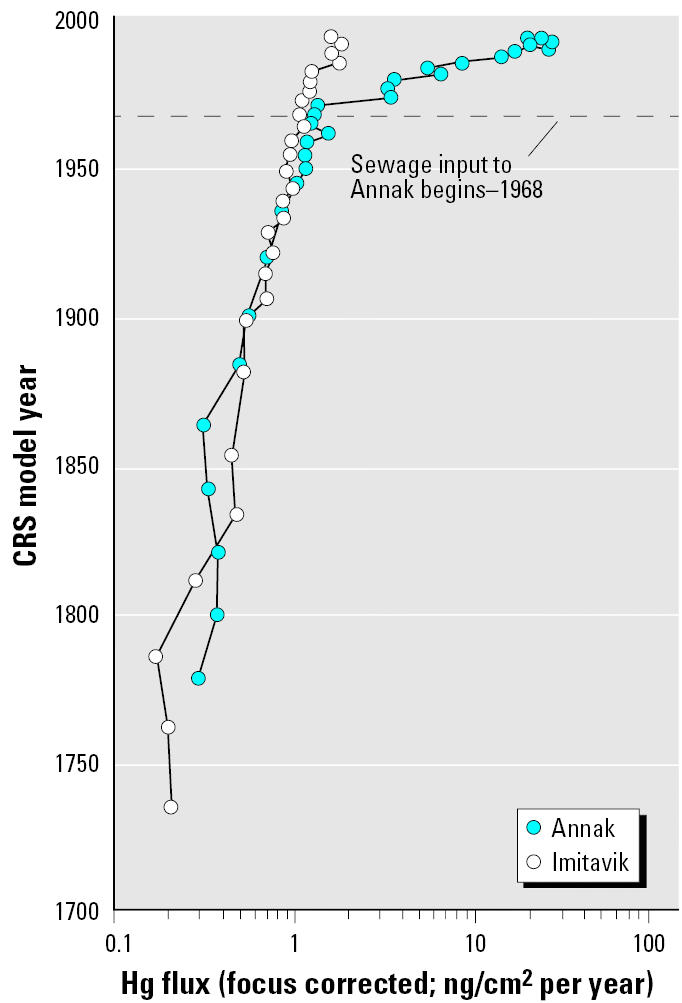
Hg inputs to Imitavik and Annak since the 1700s. CRS, constant rate of supply. Modified from Hermanson (1998).

Preindustrial Hg inputs were about 0.2 ng/cm2 per year (Figure 5). Between 1800 and 1867, inputs increased about 2-fold, and then about 3-fold by 1980. Between 1980 and 1990 the input grew 44%. These growth factors are consistent with observations elsewhere in the Canadian Arctic (Lockhart et al. 1995). Input history of Hg to Imitavik has been previously reported (Hermanson 1998).

### Annak Lake.

Pb inputs to Annak were lower than those to Imitavik until about 1950 and then show a trend similar to those to Imitavik up to the 1960s (Figure 3). Annak data show accelerating Pb inputs beginning about 1970, shortly after the beginning of sewage disposal into the lake. Pb input was about 250 ng/cm2 per year in 1970 and doubled within 12 years. It doubled again by about 1989, and peaked at 1,242 ng/cm2 per year about 1991. The decline that appears in Pb and other metals from 1991 through 1993 is an indication that inputs have dropped. However, more recent cores need to be collected to identify longer term trends.

Cd inputs to Annak are identical to the Imitavik inputs until the 1950s (Figure 4). Although Cd deposition to Imitavik stopped growing between the 1940s and about 1952, the input to Annak was unchanged between about 1944 and 1948. By 1953 Cd input in Annak increased to about 5 ng/cm2 per year and by 1960 increased to about 7 ng/cm2 per year. The higher inputs from the 1960s, apparently before sewage inputs began, were also observed in an earlier investigation (Hermanson 1991). Cd is known to be released from sediments after deposition, so there is a possibility that some Cd migrated downward in the core from greater inputs after the 1970s. However, Cd release is unlikely in reducing conditions (Khalid 1980) that are characteristic of Annak bottom waters during all times of year. After sewage inputs began, Cd inputs doubled by 1980, again by 1988, and again by 1991.

Hg inputs to Annak track the Imitavik record closely from about 1880 to the 1940s. In the late 1940s the inputs to Annak appear to be slightly higher than those to Imitavik (Figure 5). Hg inputs to Annak grew faster than Pb or Cd input after the onset of sewage disposal and between 1975 and 1991 grew from 1.6 ng/cm2 per year to 30 ng/cm2 per year (Hermanson 1998).

The results for both lakes agree with data from cores collected in 1983 and 1990 (Hermanson 1990, 1993). The Imitavik data show that the Belcher Islands region is affected by LRAT of contaminant trace metals.

The atmospheric Pb and Cd inputs to these lakes and to the Belcher Islands region stopped growing in the early 1980s and then appear to have declined. But the average human exposure as measured at Annak appears to have continued growing to 1990, suggesting the presence of other sources of Pb and Cd not related to LRAT. Pb exposure in some Inuit communities results from Pb shot ingested in country foods (Dewailly et al. 2001; Johansen et al. 2004; Scheuhammer et al. 1998) and inhaled in cigarette smoke (Dewailly et al. 2001; Rickert and Kaiserman 1994), neither of which is influenced by LRAT. The drop in Pb deposition to Annak sediments after 1990 may be associated with an intended ban on Pb shot in Canada (Scheuhammer et al. 1998). For Cd, the exposure represented in Annak may be entirely from cigarette smoke, which is known to contain high amounts of Cd and which has contributed to high body burdens in other parts of the North (Benedetti et al. 1994; Rey et al. 1997). This is also unrelated to LRAT.

### Changes in daily per-person deposition of Pb, Cd, and Hg to Annak sediments.

**Figure 6:**
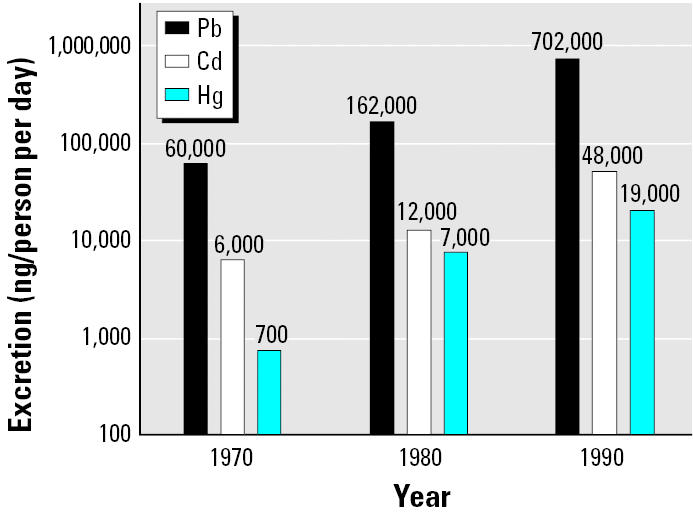
Average per capita excretion of Pb, Cd, and Hg in Sanikiluaq, 1970, 1980, and 1990.

The calculated values of person per day contribution to Annak sediment show that between 1970 and 1990 the average member of the community had growing excretion of—and apparent exposure to—all three metals, assuming that net metals deposition to Annak reflects intake and excretion (Figure 6).

The Pb value grew from 60,000 ng/person per day in 1970 to 162,000 ng/person per day in 1980 and 702,000 ng/person per day in 1990. A food study in five Canadian cities by Dabeka and McKenzie (1995) from 1986 through 1988 estimated Pb intake at 24,000 ng/person per day, considerably less than our Sanikiluaq excretion estimate. In a survey of Belgium, Malta, Mexico and Sweden, Claeys-Thoreau et al. (1987) noted a high variability in Pb excreted in feces, depending on country and its typical diet, ranging from 22,000 to 361,000 ng/person per day. Bederka et al. (1985) found an average of 180,000 ng/person per day of Pb excreted in feces in an Illinois group in the early 1980s. The results from Sanikiluaq suggest that the community has higher exposures to Pb than other groups and are generally consistent with ingestion values observed by Hansen (1990) in Greenland Inuit, which range from 69,000 to 369,000 ng/person per day and vary with meat consumption. The World Health Organization (WHO) has established a provisional tolerable weekly intake (PTWI) that calculates to a TDI for Pb of 214,000 ng/person (60-kg person) (van Oostdam et al. 1999). Values from Sanikiluaq in 1990 and from Greenland exceed this amount by > 3-fold in the worst case.

The Cd net deposition to Annak increased from 6,000 ng/person per day in 1970 to 12,000 ng/person per day in 1980 and 48,000 ng/person per day in 1990 (Figure 6). A food study in Canadian cities in 1986–1988 showed an average individual daily intake of 13,200 ng/person per day (Dabeka and McKenzie 1995). Another Canadian study by Newhook et al. (1994) showed food intake to be 12,600 for a 60-kg adult > 20 years of age. Although net Cd growth we observed in Annak sediments represents an increase of 8-fold over a 20 year period, the amounts are consistent with normal daily intake values from around the world that range from 15,000 to 79,000 ng/person per day (Kjellström 1979; Thornton 1992). They are considerably less than those in areas with known high Cd contamination in seafood, which range up to 281,000 ng/person per day (Elinder 1985; McKenzie-Parnell et al. 1988). A dietary Cd intake of 75,000 ng/person per day was considered a maximum safe level in the 1980s (Naylor and Loehr 1981), whereas the Canadian TDI in the 1990s was 60,000 ng/person per day (van Oostdam et al. 1999). The amounts observed in Annak sediments suggest that the average Sanikiluaq community member may not have been in danger of suffering from ill effects of Cd exposure from 1970 to 1990.

Hg net deposition to Annak increased from 700 ng/person per day in 1970 to 7,300 ng/person per day in 1980, to 19,000 ng/person per day in 1990, again, the fastest growth of these three metals (Figure 6). An urban Canadian food study from 1998 through 2000 found an average intake of 1,320 ng/person per day, just 7% of the Annak value in 1990 (Dabeka et al. 2003). The Canadian TDI for total Hg in the 1990s was 42,840 ng/person per day, but for methyl-Hg it was 28,260 ng/person per day for a 60-kg person, the same as that calculated from the WHO PTWI in 1999. In 2003 a Joint Food and Agriculture Organization (FAO)/WHO Expert Committee on Food Additives decreased this TDI level to an equivalent of 13,714 ng/person per day (IPCS-INCHEM 2004). In 1990 the average resident of Sanikiluaq would not have ingested or inhaled Hg beyond the TDI at the time. The recent reduction of the TDI level suggests that the community may be in the range of exposure values where ill effects may be experienced. However, the Annak values are lower than some food ingestion values in the Arctic: an Inuit food survey on Baffin Island by Chan et al. (1995) in the early 1990s showed 65,000 ng/person per day for women and 97,000 ng/person per day for men. Hg mean daily intake in Hansen’s (1990) study of four Greenland regions ranged from 25,000 to 128,000 ng/person per day and was related to meat consumption. Clearly, many Inuit Hg ingestion values exceed recent FAO/WHO limits.

## Conclusions

Our results show that after sewage disposal to Annak began in the late 1960s, inputs of Pb, Cd, and Hg from domestic sewage grew rapidly and that the community has greater exposure to Pb than to either Cd or Hg.

Between 1970 and 1990 the average exposure to each community member to Pb increased > 10-fold, Cd about 8-fold, and Hg about 27-fold. Members of the Sanikiluaq community are exposed to Pb at levels that exceed TDI levels, and Hg intake is likely also above TDIs, although those limits vary depending on the health agency that defines them. These increased exposures occurred up to 1990 despite efforts to reduce industrial and urban emissions in the mid latitudes.

Sources of metals to the community during 1970–1990 probably included the effects of LRAT, Pb from gunshot, and a considerable amount of Cd, some Pb, and Hg from tobacco smoke.

These results show that undisturbed sediments from sewage lakes can be used to estimate community exposure to contaminants if there are no agricultural or industrial inputs to the waste stream.

## Figures and Tables

**Table 1:** Estimated personal care product contributions of Pb, Cd, and Hg to Annak [ng/person per day (% of individual community member contribution in 1990)].

Personal care products	Pb	Cd	Hg
Laundry powder^a^	1,700 (0.24)	4,300 (8.9)	210 (1.1)
Cosmetics^b^	753 (0.10)	527 (1.1)	87 (0.46)
Shampoo^c^	210 (0.03)	10 (0.02)	45 (0.24)

a Assumes 16.7 g/person per day (Jenkins and Russell 1994).

b Assumes 2.5 g/person per day (Demanz et al. 1984).

c Assumes 50 mL/person per day use (LeBlanc et al. 1999).

**Table 2:** Background (Imitavik) and sewage-enriched (Annak) focus-corrected fluxes of Pb, Cd, and Hg from 1960 (presewage), 1970, 1980, and 1990 (ng/cm2 per year), Belcher Islands.

	Imitavik	Annak
Pb		
1960	180	193
1970	220	250
1980	310	426
1990	297	963
Cd		
1960	4.1	6.8
1970	4.5	7.1
1980	5.1	13.4
1990	5.1	51.2
Hg		
1960	1.1	1.5
1970	1.1	1.3
1980	1.2	6.3
1990	1.8	19.6
